# The Molecular Basis of Resilience: A Narrative Review

**DOI:** 10.3389/fpsyt.2022.856998

**Published:** 2022-05-06

**Authors:** Megan Ryan, Rebecca Ryznar

**Affiliations:** ^1^College of Osteopathic Medicine, Rocky Vista University, Parker, CO, United States; ^2^Molecular Biology, Department of Biomedical Sciences, Rocky Vista University, Parker, CO, United States

**Keywords:** resilience, hypothalamic-pituitary-adrenal (HPA axis), autonomic nervous system, neurotransmitters, immune biomarkers, stress, PTSD – post-traumatic stress disorder, cytokines

## Abstract

Resilience refers to the adaptability of a person – an ability to “bounce-back” from stressors. We question if resilience can be strengthened, potentially to decrease the risk of stress-related disorders. Unfortunately, the molecular origins of resilience are complicated and not yet well understood. In this review, we examine the various physiological biomarkers of resilience, including the associated genes, epigenetic changes, and protein biomarkers associated with resilient phenotypes. In addition to assessing biomarkers that may indicate higher levels of resilience, we also review at length the many biomarkers that confer lower levels of resilience and may lead to disorders of low resilience, such as anxiety and depression. This large and encompassing review may help to identify the possible therapeutic targets of resilience. Hopefully these studies will lead to a future where stress-related disorders can be prevented, rather than treated.

## Introduction

Resilience has been defined in many different ways, although the major theme of resilience surrounds a certain capacity to recover from stressors and traumatic events. Dr. Steven Southwick once referred to resilience as “the ability to bend but not break, bounce back, and perhaps even grow in the face of adverse life experiences.” Other authors define resilience as a “healthy functioning after a highly adverse event” (1). Further, it is believed that resilient individuals not only react to stress more adaptively, but that there is a type of resilience in which individuals create a world in which stress is less likely to occur (2). Ultimately, resilience is another word for adaptability to stressors (3). As will be discussed further, resilience is a dynamic process, and can be changed drastically throughout a lifetime due to environmental exposures and traumatic events (4).

The link between resilience and stress on a scientific standpoint remains unclear. Many research groups define resilience as the “absence of symptoms” after a traumatic event has occurred. Essentially, the examination of resilience can take place only after trauma, as a resilient individual would remain symptom-free (5). There is no doubt that there are major connections between resilience and stress-related disorders, however, it is important to recognize that resilience is not simply the absence of mental illness. Although, as examined in this narrative review, we see that many research groups examine resilience based on the absence of symptoms after trauma. As unclear as the scientific definition of resilience is, it is accepted that resilience is especially important with regards to stress. It is what allows people to cope with various stressors, and what prevents people from “breaking.” For instance, a major question in the field of resilience is why do certain military veterans suffer from PTSD, whereas some veterans do not? In this paper, we examine the various genetic, epigenetic and protein biomarkers implicated in studies of resilience. We not only examined markers of high resilience and lower stress states, but also indicators of low resilience and high stress states as well. We asked the question – what grants certain individuals the desirable trait of resilience, whereas other individuals are more prone to higher levels of stress and other mental disorders, possibly due to lower resilience?

The global burden of stress has steadily increased (especially over the past few years), gaining serious ground during the COVID-19 pandemic. One review of stress, anxiety, and depression in the general population analyzed 9,074 individuals among five studies, finding that the prevalence of stress, anxiety, and depression was 29.6, 31.9, and 33.7%, respectively (6). Another study found similar results in 398,771 participants, and also reported finding symptoms such as psychological disturbances, insomnia, suicidal ideations, and more (7). The statistics are even more staggering for individuals working in high-stress environments, such as military veterans or first responders (8). The significant impact of stress-related disorders on the world’s population itself warrants further research into what makes an individual resilient. In previous literature, stress-related disorders such as PTSD have been linked to poor resilience (9, 10). Kalisch et al. proposed that therapeutic strategies to treat stress-related disorders should begin with elevating an individual’s resilience (9). This is what makes resilience research vital for stress management. By identifying factors of resilience as well as factors of high stress, there may be an opportunity to mitigate these factors for stress treatment, or promotion of resilience, in the future.

Resilience is a multifaceted and complicated phenomenon. While there are many psychological and trait factors at play, there are also more physical indications, such as protein biomarkers, that are currently being studied (11, 12). To properly develop interventions for resilience, any factors that positively or negatively impact resilience must be thoroughly examined. This must go further than a behavioral understanding to understand the entire picture. There is currently growing research on biomarkers involved in the stress response, including biomarkers of the hypothalamic-pituitary-adrenal (HPA) axis (cortisol, ACTH, DHEA, etc.) (11) immune and inflammatory biomarkers (IL-6, CRP, fractalkine, CX3CL1, etc.) (12, 13). There are also many gene variants involved (often associated with important protein biomarkers), such as the *SLC6A4*, *5-HTTLPR*, *BDNF*, and *CHRH1* genes. The presence of these gene variants may result in either negative or positive associations to resilience. The findings surrounding these gene variants have been quite variable (14). Perhaps more importantly, many of these genes, as well as microRNAs, can be modified through epigenetics (i.e., methylation/demethylation, upregulation/downregulation, etc.), which include changes to sequences due to environmental influences (14).

Various studies show the level of impact of environmental exposures on an individual’s resilience (15, 16). Environmental factors of resilience include factors like stable relationships, higher levels of education, and supportive family environments. One meta-analysis analyzed the physical biomarkers, psychological factors, and environmental factors associated with resilience outcomes. Jaffee et al. found that individual characteristics were strongly associated with resilience only when the level of adversity in the environment was low (17). Another review focused on work-place environmental factors within nursing staff, which is considered a high-stress job. They found that a constantly negative workplace environment and culture lead to higher levels of burnout and lower resilience levels. In contrast, having a supportive workplace environment that fostered personal development and self-care was associated with a nurse’s ability to recover from environmental stressors (16). These studies represent some environmental factors that can impact a person’s resilience, representing how multifaceted resilience is.

**FIGURE 1:**
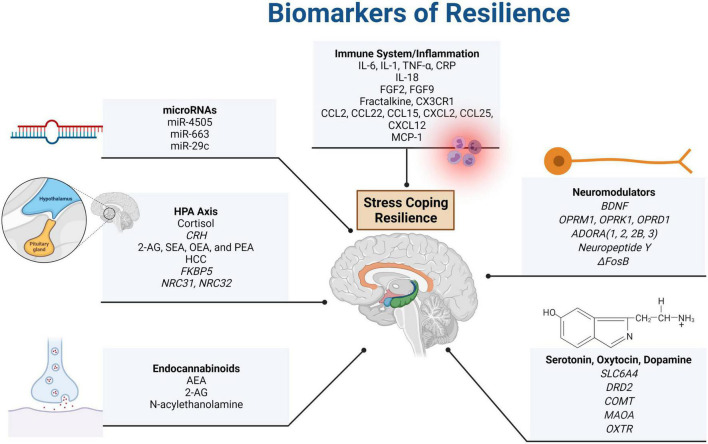
Summary of resilience biomarkers presented in this paper. Systems that were found to impact resilience included the immune system, the HPA axis, the ANS (and associated neuromodulators), as well as the endocannabinoid and endorphin (dopamine, serotonin, and oxytocin) systems. The acronyms included in the figure are defined as follows: actinin alpha 4 (ACTN4), adenosine P1 receptor (ADORA), adenylate kinase (AK4), anandamide (AEA), 2-arachidonoylglycerol (2-AG), brain-derived neutrotrophic factor (BDNF), catechol-O-methyltransferase (COMT), C-C motif chemokine ligand (CCL), complexin 1 (CPLX1), corticotropin-releasing hormone (CRH), C-reactive protein (CRP), C-X-C motif chemokine ligand (CXCL), C-X3-C motif chemokine receptor 1 (CX3CR1), dopamine receptor D2 (DRD2), FK506 binding protein (FKBP), epoxide hydrolase 4 (EPHX4), fibroblast growth factor (FGF), hair cortisol concentration (HCC), inositol-tetrakisphosphate 1-kinase (ITPK1), interleukin (IL), microRNA (miR), monocyte chemoattractant protein-1 (MCP-1), neuritin 1 (NRN1), monoamine oxidase A (MAOA), nuclear receptor subfamily C (NR3C), oleoylethanolamide (OEA), opioid receptor mu 1, kappa 1, and delta 1 (OPRM1, OPRK1, and OPRD1), oxytocin receptor (OXTR), palmitoylethanolamide (PEA), rabphilin 3A (RPH3A), SH3 domain containing GRB2 like 1 (SH3GL1), small glutamine rich tetratricopeptide repeat co-chaperone beta (SGTB), solute carrier family 6 member 4 – serotonin transporter (SLC6A4), stearoylethanolamide (SEA), tumor necrosis factor (TNF), and ubiquitin-activating enzyme E1 (UBA1). Created with BioRender.com.

While biomarkers of resilience have not yet been thoroughly studied, there is a large amount of evidence for psychological protective factors of resilience, which has been highly associated with environmental factors (18–20). Some protective factors early in life include self-regulation, family support, school support, and peer support (18), while social support and self-care (i.e., exercise, sleep, and work-life balance) are highly important throughout the entire life (19, 20). Major psychological therapies and resilience training incorporated in the past that have had a positive effect on individual resilience include cognitive therapy and mindfulness training. For example, mindfulness training has been shown to cause a significantly greater drop in adrenocorticotropin hormone (ACTH) and pro-inflammatory mediators (markers of resilience discussed below) of the immune system during stressful tasks, showing potential that mindfulness training may result in a heightened ability to cope with stressors (21). There is also promise shown with resilience and stress-inoculation training that many first responder academies have already incorporated into their curricula (22). Reviewing some of the physical biomarkers of resilience, including genetics, epigenetics, and protein biomarkers (including neurotransmitters and their receptors), will give a more complete picture that could lead to more individualized medical therapeutic interventions in the future. All factors reviewed are included in Figure 1.

Identifying the genetic components of resilience is a difficult task. Simply providing evidence that there are genetic aspects to resilience has been a challenge. Many studies have sought to provide evidence for the heritability for resilience. One representation of the genetic component of resilience is through longitudinal twin studies – utilizing monozygotic and dizygotic twins. Through the use of questionnaires given to 7,500 twins, Amstadter et al. assessed stressful life event exposure and associated internalizing symptoms (i.e., depression, anxiety, and sleep difficulty) at two different time points (23). The results of the questionnaires represented an equal representation of heritability and environment (adverse events and time) on the construct of resilience, further finding that the “stable component” of resilience was approximately 50% heritable. Specifically, when measuring only the genetic (or stable) indicators of resilience among the twin population, Amstadter et al. discovered that genetic and environmental indicators of resilience were almost equal in impact (23). Neuroticism showed the largest genetic and phenotypic relationship with resilience when reported on the questionnaires, representing a negative correlation to resilience and increasing risk for internalizing disorders (i.e., generalized anxiety disorder) (24). Similar studies have been performed, including a study specifically in a female twin sample (*N* = 2,056) that found 11% heritability in perceived resilience (25). In contrast, a male twin study among 3,318 male twin pairs in the Vietnam Era Twin Registry found a 25% heritability of resilience (as well as a 49% heritability of PTSD) (26). Showing the relationship between heritability and environment is the first step in studying the genetic components of resilience, as it evidences that there is indeed a genetic background to resilience.

Epigenetics is an up-and-coming topic in the past few decades, especially regarding resilience. Epigenetics refers to heritable information that is separate from the DNA sequence, and can be modified by environmental exposures. More specifically, this non-DNA sequence information refers to chemical modifications to DNA bases or histone proteins that result in upregulation, or downregulation of a gene, in response to a stressor. Changes in the environment can impact resilience status through epigenetics. Epigenetics provides evidence that resilience can be altered through one’s lifetime due to environmental changes. A good example of the epigenetic involvement in resilience would be the methylation of *FKBP5* intron 7 in Holocaust survivors, which appears to be passed down in their children (although it’s been shown that their children have lower levels of methylation) (27). As will be discussed further, the methylation of *FKBP5* (a gene involved in the HPA axis), is actually a protective mechanism against extreme stressors and trauma, working to decrease a probable hyperactive response to stress. DNA methylation at CpG islands results in silencing of the downstream genomic region. Consequently, it would appear that epigenetics (as occurring through environmental triggers or heritability) can cause higher levels of resilience as a protective mechanism that allows humans to adapt to stressful situations. Some epigenetic changes associated with resilience will also be mentioned in this review.

Under the major topics of genetics, epigenetics, and protein biomarkers, there were many major systems identified as having significant roles in resilience. These systems include the hypothalamic-pituitary-adrenal (HPA) axis, various neuromodulators, the autonomic nervous system, enkephalins and opioid receptors, the serotonergic and dopaminergic systems, the immune systems, and the endocannabinoid system. Further examination of markers in each of these systems and their role in resilience is needed. For now, a summary of findings of these markers can be found further in this review.

### Studies Into the Genetics of Resilience: Genome-Wide Analyses

An advancement in the study of resilience heritability is through the use of genome-wide association studies to examine the genetic relationships. By mapping the entire genome of individuals, researchers are able to identify similar genes across conditions, such as resilience or stress-related disorders. As an example, one study utilized blood samples to map the DNA genomes of 3,494 combat-exposed United States Marines and Sailors that met the PTSD DSM-IV diagnostic criterion (28). The study did not identify genes related to resilience, rather, the lack thereof in PTSD ensuing post-combat. A review on genome-wide analyses defined lack of resilience as greatly increasing risk for PTSD and other stress-related psychiatric disorders, such as major depressive disorder (MDD) (29). The thought was that a lower level of resilience predisposed these soldiers to various stress-related illnesses. One particular gene identified in a few genome-wide analysis studies, *PRTFDC1*, is a novel gene in the study of resilience. It codes for phosphoribosyl transferase containing 1 and belongs to the purine/pyrimidine phosphoribosyltransferase family. It has been postulated that it could be involved in purine ribonucleoside salvage, which is especially important for the resynthesis of adenine nucleotides and RNA in the brain. However, while *PRTFDC1* contains a phosphoribosyl transferase domain, it lacks conservation of certain residues critical to its postulated function (30). It has also recently been identified as a possible tumor suppressor gene in the diffuse tissues it’s expressed in (28). The allele rs6482463 is now being recognized as a possible predictor of stress vulnerability and low resilience (28, 29).

Another genome-wide analysis performed on 11,492 United States Army soldiers identified alleles of *DCLK2* and *KLHL36* (rs4260523 and rs12716755, respectively) in DNA taken from blood samples that were associated with self-assessed resilience per the questionnaire provided (31). The soldiers took a questionnaire pre-deployment to Afghanistan, and again 3- and 9- months following return. Resilience was self-assessed via various components, such as the ability to keep calm during a crisis, manage stress, and get along with people (connection). They were also rated on exposure to stress and trauma during deployment. Interestingly, one of the identified genes, *DCLK2* (encodes for Double Like Cortin Kinase 2), is a neighboring gene to *NR3C2*, which is a mineralocorticoid receptor gene. The authors postulated that *DCLK2* might be involved in modulation of the *NR3C2* gene, which has been associated with stress resilience in the past. The *DCLK2* variant identified was strongly associated with positive social skills, which is identified as a component of resilience. The SNP in *KLHL36* (Kelch Like Family Member 36) also had a significant association with self-reported resilience, and this variant has previously been discovered as a risk variant for late onset Alzheimer’s Disease. This gene ubiquinates proteins as part of their degradation pathways and is widely expressed (31). While these are just a couple of examples of genes that were uncovered from a genome-wide analysis, we discuss a variety of genes that have shown a strong association with resilience.

### A Major Discussion in the Epigenetics of Resilience: MicroRNAs

MicroRNAs are small non-coding RNA sequences that have major roles in gene expression, therefore, they play a significant role in epigenetics. MicroRNAs inhibit mRNA translation, especially in the brain, which means they have a role in neurodevelopment, neurogenesis, and synaptic plasticity. Recent research has shown a possible role in resilience, with exposure to stress or traumatic events activating the microRNAs in a way that confers resilience (32). In addition, microRNAs themselves can be affected by epigenetics – through methylation, histone acetylation, chromatin remodeling, etc. As of late, there is more research regarding microRNAs in mice and rats (due to the inability to access brain tissues in living humans), so their role in humans is a novel investigation.

There is increasing evidence for the role of microRNAs in resilience and psychiatric disorders. Current human studies of microRNAs need to use alternative methods – assessing microRNA expression in the blood or through studies of genes encoding miRNA biogenesis-related proteins. Post-mortem studies have also been conducted, but for obvious reasons they are not able to collect as much data and dead brain tissue is not as reliable as live. For example, in patients with milder symptoms of anxiety disorder (as assessed by the Hamilton Anxiety Scale), there were higher levels of circulating miR-4505 and miR-663 in blood samples (33). In addition, a prior study found that miR-29c expression (as shown by genotyping lymphocytes in peripheral blood samples) was increased in individuals exposed to stress through a serial subtraction arithmetic task (34). Interestingly, microRNAs such as this don’t simply have a role in stress resilience, but also physical resilience. For instance, miR-29c has also been shown to correlate with survival rate in breast cancer through its inhibition of certain genes involved in the pathogenesis of breast cancer (35). miRNAs are an exciting new target of resilience studies and new methods of identifying their association with resilience in humans must first be discovered.

## Hypothalamic-Pituitary-Adrenal (HPA) Axis Involvement in Resilience

The HPA axis is a major modulator of our response to stress. While the autonomic nervous system is our body’s immediate defensive system to stress, the HPA axis produces a slower response through the release and action of glucocorticoids. The glucocorticoids, such as cortisol, bind to steroid receptors in the brain to affect the expression of genes, ultimately modulating our behavioral reaction and promoting a pro-inflammatory state after exposure to stressful events. Previously in the literature, it has been demonstrated that changes to this system can have both positive and negative effects on resilience (36). In fact, past research has shown that glucocorticoid levels are elevated in a variety of stress-related disorders, such as major depressive disorder (MDD) (36). Glucocorticoids in the HPA axis, such as cortisol, have been thoroughly studied on the subject of stress resilience, however, the role of the genes themselves have yet to be further explored. We will discuss the literature regarding a few of the HPA-axis-related biomarkers indicated in resilience.

The ultimate result of the HPA axis in response to stress is to stimulate CRH release from the hypothalamus, which stimulates the pituitary to release ACTH. This causes further release of cortisol and the neurosteroid dehydroepiandrosterone (DHEA) (23, 24). When cortisol binds to GRs, there is a negative feedback inhibition of the HPA axis. DHEA has been shown to suppress this action of cortisol (37). Cortisol and DHEA are of interest due to their important roles in the HPA axis, as a high DHEA:cortisol ratio has been associated with reduced stress-induced dissociative symptoms in military members. In addition, DHEA has antidepressant and anxiolytic effects (38). It has also been shown to grant higher levels of self-care, and overall resilience, in previous studies (39). Many epigenetic changes have also been shown to change the way the HPA axis can respond to stress, including DNA methylation to dampen the response of the HPA axis (40). Epigenetic effects that alter the HPA axis and result in changes to levels of cortisol and DHEA could result in changes in levels of resilience. At the protein level, a study conducted on 120 healthy male participants exposed to a stress-induction and control procedure, found that high basal salivary cortisol levels were related to relative stress resilience reflected by higher extraversion scores and an overall lower stress induced increase in amygdala activity (41). Inconsistent associations have been reported with regards to trauma experienced and changes in cortisol secretion. Some studies show individuals who have experienced trauma have lower overall cortisol levels, but others suggest that trauma can lead to hypersecretion (42). For example, a previous study investigating salivary cortisol levels in 733 war affected adolescents undergoing an 8 week intervention identified fear or insecurity as being predictive of a trajectory of increased cortisol production (hypersecretion), specifically with every one percent increase in levels of insecurity, adolescents were 0.02 times more likely to have a trajectory of hypersecretion (43). Higher hair cortisol levels have also been correlated with heavier workload in 72 German EMS personnel (44). Overall, it should be noted that cortisol levels after trauma/stressful exposures are highly variable and individualized. The impact of cortisol levels on resilience is also dependent upon the environmental condition. DHEA and cortisol have long been topics of stress, but these studies suggest a powerful association with resilience that needs to be further examined.

### Corticotropin-Releasing Hormone Receptor

Corticotropin-releasing hormone (CRH) is produced by the hypothalamus. After release, it activates adrenocorticotropic hormone (ACTH). ACTH stimulates the synthesis of cortisol, mineralocorticoids, and DHEA. A gene that has been implicated directly in stress resilience is *CRHR1*, which codes for the CRH receptor (45). There are several genetic variants of *CRHR1* that have been associated with either lower or higher resilience levels. One such variant, the G-allele of rs878886, was identified by Sleijpen et al. in their examination of refugee and Dutch adolescents’ blood samples. The G-allele was associated with lower levels of resilience and was connected with self-reported dissatisfaction with life, especially when compared with the C/C homozygotes (45). *CRHR1* may offer protective effects for adolescents who have experienced trauma. Furthermore, in adults exposed to hurricanes, *CRHR1* alleles rs12938031 and rs4792887 identified in DNA samples from saliva were associated with dysregulation of the HPA axis, low resilience, and development of PTSD symptoms. rs12938031 was associated with a firm diagnosis of PTSD post-hurricane (evaluated via phone interview 6–9 months after hurricane exposure) (46). Certain other variants/polymorphisms of *CRHR1* are associated with a reduced risk of depressive symptoms and resilience in children exposed to early life stress (29). These findings support a protective role of *CRHR1* in resilience. Unfortunately, there are not many discussions of this receptor in regards to epigenetics or its levels in the body, but examination of the gene and associated alleles shows promise.

### FK506 Binding Protein

*FKBP5* gene is another highly significant gene in the HPA axis, encoding for the FK506 binding protein 51 (FKBP5) (29). FKBP5 is a heat shock protein co-chaperone that interacts with the glucocorticoid receptor to inhibit its activity, overall resulting in a decreased negative feedback inhibition of the HPA axis. In past research and gene × environment studies, variants of the *FKBP5* gene have been used to predict PTSD probability in the presence of childhood trauma and are associated with an increased risk of developing other stress-related disorders, such as anxiety and depression (29). Through the use of clinical interviewing and questionnaires as well as blood sampling, a longitudinal study demonstrated that the T-allele of the *FKBP5* rs1360780 polymorphism delayed cortisol recovery after exposure to acute psychological stress (giving an oral presentation), causing HPA axis dysregulation. In contrast, the CC genotype had faster cortisol recovery after acute stress in young adults who had experienced maltreatment during childhood (47). This possibly indicates a protective role of the CC genotype, compared to lower levels of resilience in the TT genotype. Indeed, it also demonstrates gene × environment interactions in both groups. An additional study discovered an additive effect of *FKBP5* variants when paired with variants in the *CRHBP* (corticotropin releasing hormone binding protein) gene (48). When children with these variants were exposed to childhood trauma, their risk of suicide was greatly increased, further demonstrating the complicatedness of gene interactions in resilience.

While SNPs and specific alleles of the gene could have different results on resilience (i.e., the protective C allele of FKBP5 rs1360780 has been shown to increase DHEA levels), epigenetics is also an important regulator of this gene’s role in resilience (49). In a study conducted on the rs1360780 polymorphism, saliva samples were analyzed for methylation of the *FKBP5* gene. Individuals who self-reported significant early trauma exposure in their life (moderate-to-severe physical, emotional, or sexual abuse), and who carried the T risk allele of rs1360780, had demethylation of intron7 CpGs. In the protective C allele in individuals who had not experienced significant trauma, methylation levels remained stable (50). The researcher group hypothesized that the demethylation, and thus activation, of the protective C allele better allowed individuals to increase their stress response. Overall, the demethylation not only increases sensitivity to stress, but also results in changes in immune cell gene expression and other carry-down effects (50). However, this is in contrast to what has been shown in Holocaust or World Trade Center Survivors – whose extreme levels of trauma actually resulted in the methylation and decreased expression of *FKBP5* (27, 51). Researchers of those studies thought that the dampening of the stress response through decreased expression of *FKBP5* actually helped to make those individuals more resilient. Whether methylation to dampen the response versus demethylation to increase sensitivity to stressors results in higher levels of resilience needs to be investigated more. This gene is an important example of the disagreement on the topic of resilience currently.

### Glucocorticoid and Mineralocorticoid Receptors

The *NR3C2* gene encodes for the mineralocorticoid receptor (MR) involved in the HPA axis, predominantly expressed in the hippocampus and amygdala (52). In response to binding of corticosteroids (especially cortisol), the mineralocorticoid receptor increases the transport of sodium and potassium across cells. It can also aid in an increased release of inflammatory mediators of the immune system. The receptor is an important mediator of stress regulation and has been implicated in resilience. In a past study of resilience associated with negative memory formation including 483 participants, it was demonstrated that homozygotic A-carriers of rs5534 variant of *NR3C2* had increased formation of negative memory bias, especially when exposed to traumatic life events. Other polymorphisms of *NR3C2* have been associated with disorders such as depression and PTSD (52). The dysregulation of MR expression caused by the variant and adverse life events together may indicate that proper function of the *NR3C2* gene and MR is needed for optimal resilience. Another study involving 193 adolescent girls (76 exposed to emotional trauma), showed that those with the G-allelic variant, identified in saliva DNA samples, and had been exposed to trauma had significantly smaller left hippocampal volumes, as shown by magnetic resonance imaging (MRI) scans (53). The variant itself was attributed to the change in hippocampal size, as those without the G-allelic variant did not have as significant of hippocampal volume change. A smaller hippocampus is associated with stress-related disorders (although this effect is not yet well understood), therefore, certain variations of *NR3C2* may accord lower resilience against these disorders.

Methylation of the *NR3C1* gene is associated with stress exposure in childhood (54). In a study of 534 school-aged children (285 maltreated and 249 non-maltreated children as assessed by the Maltreatment Classification System), children were asked to participate in camp activities for 35 h a week and provide salivary DNA samples. During camp activities, research assistants observed the children and then completed measurements such as the Emotional Regulation Checklist and the Revised Child Manifest Anxiety Scale. Children also self-reported any depressive symptoms. Results indicated significant hypermethylation of the *NR3C1* exon 1F in the maltreated child group when compared to the non-maltreated children. Overall, silencing of *NR3C1* was related to negative outcomes such as higher levels of depressive and externalizing symptoms (54). This is also seen in past literature, with methylation and dysregulation of the *NR3C1* gene contributing to psychopathology and lower levels of resilience in children (55). In addition, another prior study found that increased expression levels of the glucocorticoid receptor (GR) gene, *NR3C1*, correlated positively with chronic daily stress and the receptor has been postulated to be a protective biomarker of stress (56). Overall, proper function of this gene, and the associated receptor, is required for the stress response and for higher resilience levels.

## Various Neuromodulators and Their Role in Resilience

The autonomic nervous system (ANS), particularly the sympathetic component, is one of the most important pathways involved to the stress response (57). It is activated in states of stress to create the “fight or flight” response. In response to stressful situations, the hypothalamus secretes corticotropin-releasing hormone, which activates noradrenergic centers in the brainstem and spinal cord. The resulting release of norepinephrine and epinephrine from the adrenal medulla activates adrenergic receptors that increase blood glucose, raise the heart rate, and constrict peripheral blood vessels to spare blood flow for the heart (57). Since the ANS makes up arguably the most important part of the stress response, proper modulation of the ANS is needed for resilience. Several genes of this pathway possibly involved with resilience have not yet been thoroughly studied, such as the adenosine receptor genes and neuropeptide Y.

### Adenosine Receptor

There is a growing amount of evidence that adenosine receptor genes such as *ADORA1, ADORA2, ADORA2B*, and *ADORA3* have a neuroprotective role in various psychiatric diseases (58). Certain adenosine receptors are involved with muscle activation and pressor responses in the sympathetic pathway. These receptors are also associated with modulating neurotransmitter release, including in the mechanism of sleep to slow neural activity down (59). It has also been postulated that the adenosine receptor has a relationship with the HPA axis through its positive regulation by corticosteroids, although all of the exact functions of the adenosine receptor and associated genes have not yet been fully identified (59). Overall, the genes have important roles in several different functions, such as sleep, pain regulation, cardiac function, and more. The increased bioavailability of adenosine receptors by the adenosine receptor genes via the HPA axis could be involved in protecting hippocampal cells from injury induced by chronic stress. In particular, polymorphisms in *ADORA2A* were shown by positron emission tomography (PET) to increase A1AR availability in brain regions such as the superior frontal gyrus, the dorsolateral prefrontal cortex, and the hippocampus, where these receptors modulate the release of neurotransmitters such as glutamate and GABA. The adenosine receptor A1AR (important in the sympathetic pathway) is upregulated after negative physiological conditions like prolonged wakefulness, acute exposure to ethanol, and in responses to stress. Therefore, polymorphisms in *ADORA2A* could play a protective role in resilience (59).

### Neuropeptide Y

Neuropeptide Y (NPY) is an important sympathetic neurotransmitter, and is also indirectly involved in the HPA axis by counteracting the effects of corticotropin-releasing factor (29). Through this method, NPY prevents over-activation of the stress response. One of its main functions in the nervous system is to stimulate food intake, especially carbohydrates. Specifically in the sympathetic nervous system, it is found in adrenergic vasoconstrictor neurons, where it potentiates norepinephrine-mediated peripheral vasoconstriction in response to stressors (29). Polymorphisms of the *NPY* gene, particularly the T allele of the rs16147 polymorphism, showed consistent levels of resilience in low, moderate, and high levels of trauma exposure in 1,140 earthquake survivors and 2,370 company employees and university students (60). Gan et al. defined resilience as “the ability to overcome challenges and threats or the results of adapting oneself to adversity successfully,” and therefore measured resilience using positive future focus (60). Positive future focus is one’s ability to shift attention from the past to the future. The increase in positive future focus associated with the T allele of the rs16147 polymorphism indicates a possible increase in resilience for T allele homozygotes (60). This aligns with what others have identified in the literature – T allele homozygotes of the rs16147 polymorphism have higher levels of resilience and ability to adapt, and tend to develop higher positive focus, whereas the C allele is associated with anxiety and depressive symptoms after experiencing childhood adversity (29). A systematic review and meta analysis showed that participants prescribed psychotropic medications had significantly higher levels of NPY. This effect was unrelated to the medications, rather, the authors postulated that the medications themselves may help to restore basic levels of NPY in these patients. The review discussed that overall, NPY levels were significantly lower in plasma and cerebrospinal fluid in PTSD patients versus controls and that patients with MDD had significantly lower levels of NPY in plasma compared to controls, but not in the CSF. In addition, chronic stress patients had significantly higher plasma NPY levels than any of the other groups (61). In a randomized control trial with 24 individuals, it was discovered that a single dose of neuropeptide Y was well tolerated up to 9.6 mg and showed signs of anxiolytic effects (62). These studies indicate that a balance must be achieved with NPY in plasma. In addition, genetic studies reveal that the T-allele of NPY holds a protective effect against stress states.

### Neuropeptide S

Another neuropeptide possibly involved in resilience mechanisms is Neuropeptide S (NPS). While it has been mostly studied in rats and mice, it represents another possible treatment option for non-resilient phenotypes (if we consider higher levels of anxiety to be lower levels of resilience). The *NPSR* gene encodes for the Neuropeptide S receptor, a G-protein coupled receptor that binds to Neuropeptide S, resulting in increased intracellular calcium and cyclic adenosine monophosphate (cAMP). The entire pathway has been shown to result in anxiolysis, or decreased anxiety and sedation (63). In past research, a single nucleotide polymorphism (SNP) in the human *NPSR* gene located on chromosome 7p14-15 revealed a possible panic disorder susceptibility in DNA blood samples. Further studies confirmed an association between *NPSR* polymorphisms and anxiety disorders (63). In one specific study using 475 healthy volunteers, questionnaires on childhood trauma, anxiety sensitivity, and life threatening experiences were used, as well as blood samples, revealing a significant gene and environmental (G × E) effect of the *NSPR* A/T polymorphism (rs324981) on anxiety sensitivity. In addition, the T risk allele, or a high number of maltreatment experiences in childhood, was associated with higher levels of anxiety and an increased risk of panic disorder (64). While the neuropeptide S gene and receptor have not been directly linked with resilience, these interesting results show that this gene should be a target for future resilience studies regarding its role in stress-related disorders.

### Brain-Derived Neurotrophic Factor

The *BDNF* gene is a neuromodulator gene that encodes for the brain-derived neurotrophic factor responsible for aiding in long term potentiation and neuronal plasticity. Its effects have long been studied in literature regarding memory. With an increase in understanding of resilience, researchers have shifted their focus to how *BDNF* may have a role resilience and psychiatric disorders. In the past decade, the Val66Met polymorphism has been strongly identified as a possible genetic factor of resilience (29, 64). This polymorphism has revealed an increased risk for PTSD, as well as the severity of PTSD symptoms (29). After applying The Resilience Scale and the Hamilton Rating Scale for Depression to 106 major depressive disorder patients (at baseline, post-treatment, and at 6 months of follow-up), Peters et al. found the Met allele in DNA blood samples to have a strong association with increased resilience scores and decreased depressive symptoms (65). Especially when compared to patients with the Val/Val genotype, the Met allele showed even higher resilience in follow-up after cognitive therapy (demonstrating evidence for gene × environment interactions with the *BDNF* gene). Lower resilience in the Val/Val genotype when compared to the Met allele (Val66Met polymorphism) in male carriers was confirmed in a study of 167 male college students and the Connor-Davidson Resilience Scale (66). Conversely, a study of 116 children exposed to Hurricane Ike evaluated for measures of hurricane exposure and stress, as well as symptoms of PTSD and depression, revealed increased symptoms of PTSD and depression with greater stress exposure in those that had the Met allele (67). There are varying effects of the Met allele on resilience, especially when considering gene × environment effects. With the disagreement on the overall effect, the *BDNF* gene must be more thoroughly studied before its role in treatment can be elucidated.

## Enkephalins and Opioid Receptors

An emerging group of neurotransmitters in the study of resilience are the enkephalins. Enkephalins are neurotransmitters that act on opiate receptors to inhibit the release of further neurotransmitters such as substance P, vasopressin, and ultimately results in the increase of dopamine. The analgesic effects of enkephalins are what caused scientists to first question their potential role in resilience and anxiety (68). While genes for the group of enkephalins themselves have not yet been studied in humans, the opioid receptor genes are of particular interest in genetic studies of resilience. Therefore, in our discussion we will focus on the human studies involving opioid receptors.

There are three important opioid receptors for a discussion on resilience: mu (*OPRM1*), kappa (*OPRK1*), and delta (*OPRD1*) opioid peptide receptors. These receptors are both extensively expressed in the brain and it has been proposed that the possible modulation of these receptors could serve as one treatment in anxiety-related disorders and low levels of resilience (68). When enkephalins and other peptides bind to these receptors, they modulate activity between brain nuclei, essentially contributing to the formation of pleasant or unpleasant mood or affect. In a large study analyzing single nucleotide polymorphisms (SNPs) of these receptors, considering a positive and negative affect scale (PANAS), there were several polymorphisms identified with an association to change affect or mood (69). In assessing positive versus negative affect, a questionnaire was employed that asked questions such as “how often did you feel nervous in the past four weeks?”. These questions covered acute and chronic time periods. Higher levels of resilience would belong in the positive affect category. Masih and Verbeke identified a significant negative association between the rs1799971 and rs17174794 *OPRM1* polymorphisms and negative affect, and a positive association between the rs540825 and rs62638690 *OPRM1* polymorphisms and positive affect. In addition to the *OPRM1* receptor, both polymorphisms identified for *OPRD1* negatively correlated with negative affect, and both polymorphisms for *OPRK1* positively correlated with negative affect. This makes sense, considering that *OPRK1* actually results in a decrease in dopamine levels, whereas *OPRD1* activation has previously been shown to help reduce chronic stress and anxiety (69).

In a more recent study on the role of opioid receptors in behavior, a computer task (a game involving a computer malfunction) was used to cause frustration in a group of 62 people (70). When participants’ saliva was sampled for genotyping (the researchers selectively targeting the A118G mutation), it revealed that carriers of the A118G mutation of *OPRM1* (the mu receptor) had a slower rate of behavior recovery after experiencing frustration in the computer task, tended to abandon the task earlier, compared to participants without the mutation (70). The higher levels of frustration could easily be linked to lower levels of resilience when considering that tolerance to stress, frustration, as well as mood itself, are large components of an individual’s resilience level. The results described here for opioid receptors reveal an exciting possibility in the study of resilience. Evidence for their role in mood and affect is a great indication for future targets of treatment. Further exploring the role of enkephalins and other opiate peptides would also be an important area of study.

## Serotonin, Dopamine, and Oxytocin

### Serotonin

Serotonin (5-HT) is a neurotransmitter produced from the amino acid tryptophan and released from the raphe nuclei in the midline of the brainstem. 5-HT has many effects, including modulation of mood, anxiety, temperature, sleep, libido, and appetite (71). Overall, the ability of 5-HT to modulate our response to dress could have an impact on resilience (72). The protein product of the *SLC6A4* gene, one of the most studied genes in resilience, is responsible for the reuptake of 5-HT into presynaptic neurons, where 5-HT exerts its biological activities (71). A study performed by Amstadter et al. involved collecting DNA samples from saliva in order to specifically study the *5-HTTLPR* polymorphism of the *SLC6A4* gene (71). This polymorphism has been included in numerous research studies of resilience. Amstadter et al. had participants complete The Behavioral Indicator of Resiliency to Distress (BIRD), a challenging computerized game that is meant to cause frustration, and then they completed the PANAS questionnaire. Low levels of resiliency was represented by high distress intolerance, or quitting the game early. The results showed that the *S* allele of the *5-HTTLPR* was associated with increased likelihood of quitting the BIRD task early. Interestingly, the *S* allele is also associated with less efficient serotonin uptake (71). It should be noted, however, that other studies have identified the *S* allele as a factor of increased resilience, and the *L* allele as a risk allele for affected resilience (72). This indicates a need for more studies comparing the phenotypes of carriers of the *S* and *L* alleles to determine the exact mechanism of the serotonin gene within resilience.

### Dopamine

In indirect relation to the serotonergic pathway, dopamine is also an important piece of the resilience puzzle. Decreased serotonin leads to increased dopamine, and vice versa. Dopamine is the neurotransmitter in the brain responsible for reward, pleasure, and salience. There are four dopaminergic pathways in the brain: the mesolimbic (pleasure and reward), mesocortical (decision making and memory), nigrostriatal (motor planning), and tuberoinfundibular (libido and breastfeeding) pathways. Dopamine is produced from tyrosine and phenylalanine (both present in protein-rich foods) in response to anticipation of reward. As a result, dopamine produces a “feel-good” sensation in the brain. Significant to the discussion of resilience, dopamine can be converted to norepinephrine by dopamine beta-hydroxylase. Norepinephrine is an important mediator of the sympathetic nervous system response and our response to stress. In previous studies, dysregulation of dopamine was revealed to contribute to PTSD symptoms (and therefore low resilience), such as poor sleep and attention (73). There are several genes associated with dopamine that are implicated in studies of resilience, including, but not limited to, *SLC6A3*, *DRD2*, and *COMT*. The *DRD2* gene codes for the dopamine receptor DRD2, which helps with the synthesis and release of dopamine. The *SLC6A3* gene codes for the dopamine transporter, involved in dopamine uptake within neurons. In a systematic literature review regarding the dopamine pathway and PTSD risk, several studies identified a higher PTSD susceptibility with the rs1800497 polymorphism in *DRD2* and the VNTR in *SLC6A3*. Both of the polymorphisms have been associated with other neuropsychiatric disorders as well (73). Recent research on these genes themselves is lacking, however, they have recently become important in studies of epigenetics.

### Catechol-O-Methyltransferase

*COMT* is also of high importance in the study of resilience. The gene codes for Catechol-O-methyltransferase, an enzyme that degrades catecholamines such as norepinephrine, epinephrine, and dopamine. In our exploration of the autonomic nervous system’s relation to resilience, we briefly discussed the sympathetic nervous system. Norepinephrine is a regulator of the sympathetic nervous system, increasing heart rate, constricting peripheral blood vessels, and more during the response to stressors. *COMT* is not only important for this pathway, but also for the dopamine pathway. Polymorphisms of this gene have been of interest, such as the Val158Met (rs4680) polymorphism. A previous study utilized the Childhood Trauma Questionnaire (CTQ) and Traumatic Events Inventory (TEI), as well as the Connor-Davidson Resilience Scale, in order to assess resilience in a group of participants undergoing whole-body functional magnetic resonance imaging (fMRI). DNA was assessed via saliva. It was found that in *COMT* Val/Val carriers, childhood trauma was associated with increased inhibition related hippocampal activation, and this hippocampal activation was suggestive of a relationship between childhood trauma and higher resilience. In the Met carriers, more childhood trauma was associated with decreased hippocampal activation (74). This is interesting because the Val158Met polymorphism has actually been shown to decrease activation of the COMT enzyme, resulting in higher dopamine levels. The researchers postulated that higher dopamine levels than normal in response to stress is a negative mechanism, as it disturbs the dopamine homeostasis.

The Met allele being associated with lower levels of resilience is in contrast with what Amstadter et al. found in their distress intolerance study. They saw that Val carriers of the Val158Met *COMT* polymorphism were actually more likely to quit the frustrating task when compared to the Met homozygotes. Once again, they concluded that this could be due to increased dopamine release associated with the Met allele, however, they believed this was actually protective against stress conditions (71). These results indicate the complexity of this gene and the need for more study, while considering the effect of multigenic interactions, such as the study conducted by Kang et al. on 321 college students. Their findings showed an interaction between *COMT* and *BDNF*, such that in males with the *COMT* Val/Val genotype, students with the Val allele of *BDNF* had lower resilience than those with the Met allele. This was in contrast to males with the *COMT* Met genotype, where the Val allele of *BDNF* actually had higher resilience (66).

### Monoamine Oxidase A

The last gene involved with serotonin and dopamine metabolism that will be discussed is the *MAOA* gene. This gene provides instructions for making the monoamine oxidase A enzyme, an enzyme involved in breaking down monoamines released by neurons and glial cells, such as dopamine, serotonin, and norepinephrine. Therefore, like *COMT*, *MAOA* is involved in not only the sympathetic response to stress, but also our dopamine and serotonin responses. In the past, *MAOA* variants have been associated with aggressive and antisocial behavior, particularly in males (75). In a longitudinal study on three *MAOA* variants [*MAOA-H*, *MAOA-H/L* (heterozygotes), and *MAOA-L*], 399 teenage Syrian refugees had their saliva sampled and took various questionnaires on stress and trauma levels (such as the Lifetime Exposure to Traumatic Events and the CYRM12 Resilience measure). Results showed that *MAOA* variants had a significant association to perceived psychosocial stress. In addition, *MAOA-L* male carriers had sharper reductions in perceived psychosocial stress over time than the *H* carriers. While the *MAOA* alleles by themselves did not have a significant relation to resilience, it was found that *MAOA-L* males with either low trauma exposure or high resilience levels had the sharpest reductions in perceived psychosocial stress (75). The results of that particular study represent a strong gene × environment interaction for *MAOA*. These gene × environment interactions should be further studied for *MAOA*.

### Oxytocin and OXTR

Oxytocin, like dopamine and serotonin, is considered one of the “happy hormones,” or hormones that serve as mediators of positive affect. Oxytocin is produced by the hypothalamus and released by the pituitary gland, and it is involved with more physical actions of the body, by stimulating uterine contractions in childbirth and playing a huge role in breast-feeding. It is also involved in more emotional reactions due to its association with feelings of lust, love, empathy, etc. Oxytocin receptors are found in the ventromedial hypothalamus, ovaries, testes, and adrenals (76). A huge role of oxytocin in resilience is through its coordination of the action of the HPA axis and release of CRH (77). Dysregulation of oxytocin or its receptor (OXTR) could be associated with lower levels of resilience, and vice versa. For instance, in a GWAS of 2,163 veterans, in the *OXTR* polymorphism rs53576 (which results in a guanine to adenine change), the A allele (versus the G allele) was associated with insecure attachment and higher risk for PTSD. These insecurely attached individuals with an A allele were also more socially anxious than G homozygotes. The A allele of this polymorphism is classically associated with morphometric alterations of the hypothalamus and amygdala, as well as maladaptive outcomes (such as greater depressive symptomology and stress reactivity) (78).

The *OXTR* can also be epigenetically modified in association with resilience. In particular, DNA methylation of the receptor leads to its inability to carry out normal function. In one study of neuropsychiatric disorders and association to epigenetic changes of *OXTR*, methylation of Exon 1 of rs53576, thus decreased expression, was found in depressed women (79). Once again, this demonstrates that lower levels of oxytocin may be associated with lower levels of resilience. In summation, oxytocin is required for resilience.

## Immune System and Inflammation

The immune system response has long been known to modulate our response to stress, especially when considering the chronic response to stress over a long period of time. The innate immune system (involving cells such as macrophages and dendritic cells) is the immediate response to pathogens and injury. The response to these cells includes the release of inflammatory mediators such as prostaglandins, histamines, and serotonin, thus causing vasodilation, pain receptor stimulation, and release of pro-inflammatory cytokines. There is growing evidence that children who endure traumatic experiences during their childhood have elevated immune system activity (40). Dendritic cells and macrophages are responsible for releasing cytokines such as interleukins (i.e., IL-6) and tumor necrosis factor alpha (TNFα). Psychological stress has been shown to cause a reaction similar to the immune response to pathogens or injury, in that psychological stress also results in a release of proinflammatory cytokines. The adaptive immune response (involving T and B lymphocytes) is known as storage for the immunological memory of a stressor, and is also important in the study of psychological stress and resilience (80). In this section, we will discuss the various cytokine genes related to lower or higher levels of resilience, such as IL-6. We will also mention other genes associated with resilience due to their role in the stress response. It should be noted that genes of the immune response possibly involved in resilience are vast in number, however, like many other biomarkers, these genes have been more thoroughly studied in animal research.

### IL-6

Interleukin-6 (IL-6) has many different roles in the innate and adaptive immune system. It is released by monocytes and macrophages, as well as by glial cells in the brain. IL-6 causes fever, induces the release of C-reactive protein from hepatocytes, stimulates the production of the clotting factor fibrinogen, and helps T- and B-cells to differentiate (80). It also stimulates the HPA axis, which we know has an important involvement in resilience. IL-6’s many roles in the immune system have made it an easy target for resilience studies. Correlation studies examining associations between IL-6 levels and resilience have shown mixed results. Levels of IL-6 in serum samples from 299 Vietnam combat veterans indicated that PTSD severity was correlated with small but significant decreases in IL-6 whereas resilience was correlated with increased levels of IL-6 (81). Another study analyzing blood samples from women with PTSD compared to healthy controls showed significantly higher levels of IL-6 and lower resilience scores using the QOL (World Health Organization Quality of Life-BREF) scale (82). Conflicting results from these studies warrants further research in order to determine the role of IL-6 in resilience.

### Fibroblast Growth Factors

Fibroblast growth factor 9 (fgf9) and fibroblast growth factor 2 (fgf2) are also important in the HPA axis, as well as being involved in the inflammatory response to stress. *FGF9* and *FGF2* are more novel genes, however, epigenetic changes in these genes may result in changes to resilience. It’s still unclear how exactly these genes and biomarkers are involved in the stress response, but it is known that they have a regulatory effect on the HPA axis. *FGF2* expression levels are positively correlated with HPA axis activity, whereas *FGF9* is negatively correlated with *FGF2*. In a study of postmortem hippocampi gene expression, it was found that *FGF9* expression was increased in depressed individuals, whereas *FGF2* was decreased (83). It was proposed that *FGF9* expression may inflict lower levels of resilience. *FGF2*, conversely, may be indicative of higher levels of resilience, as shown by the negative correlation between salivary levels and fear expression in one study (84, 85). When looking at fgf2 expression in brain regions of individuals with depression, schizophrenia or bipolar disorder, the results are interesting. The density of fgf2 mRNA positive cells in CA4 was in depression compared to controls, but overall the fgf2 mRNA levels were higher in DG than compared to the CA1 and CA4 regions of the brain (86). An additional study showed that human participants with low salivary fgf2 exhibited significantly heightened skin conductance responses to a conditioned stimulus during a fear conditioning test, leading to the conclusion that low fgf2 is associated with an increase in anxiety in humans (84). The other fibroblast growth factors are also of interest in other resilience research, however, much of this research is still in mouse and rat stages. The fibroblast growth factors will be an interesting addition to resilience research in the future.

### Fractalkine and CX3CR1

A more recent finding of current research is the role of fractalkine and its receptor, CX3CR1 (a chemokine). Fractalkine and CX3CR1 are responsible for controlling the release of inflammatory cytokines by influencing communication between glial cells and neurons. CX3CR1 is found on the surface of microglia in the CNS. In previous studies involving mice, it was found that selective knockout of CX3CR1 gene on monocytes and macrophages promoted resilience to stress by preventing their recruitment in the mouse brain [therefore preventing the inflammatory response to stress (80)]. Very few human studies regarding fractalkine and its receptor have been conducted. In one study assessing mRNA expression of 16 glia-related genes in the dorsal lateral prefrontal cortex and the anterior cingulate cortex between patients with schizophrenia that died by suicide or died naturally, it was identified that patients who had died by suicide had higher levels of expression of the CX3CR1 mRNA (87). Another study showed that fractalkine positively correlated with anxiety and depression scores prior to receiving chemotherapy and surgery in cancer patients (88). Contradicting these findings, Zhang et al. measured chemokines in blood of United States military service members deployed to Iraq and Afghanistan, and found that fractalkine was associated with resilience to PTSD (89). Future research is needed to understand the intricacies of fractalkine involvement in building resilience, as the current studies contradict each other on the roles of fractalkine and its receptor in this topic.

### MCP1 (CCL2)

MCP-1 or monocyte chemoattractant protein-1 is a key chemokine that regulates migration and infiltration of monocytes and macrophages. It is produced by a variety of cell types by oxidative stress, other cytokines or growth factors. MCP-1 is one of the most well studied within the chemokine family and has been suggested as a potential pharmacological target for multiple diseases including multiple sclerosis, rheumatoid arthritis, atherosclerosis and diabetes (90). Recently, it was found that the salivary level of MCP-1 was significantly correlated with PTSD symptoms, depression and anxiety in hurricane survivors (91). This result was also confirmed by a study investigating salivary MCP1 levels in troops deployed to Iraq and Afghanistan who were diagnosed with PTSD post-deployment (89). Future studies need to examine the possibility of lower MCP-1 levels leading to higher resilience.

### IL18

IL18 is a proinflammatory cytokine expressed by activated macrophages and dendritic cells, that stimulates the release of other cytokines from these cells. It is expressed widely throughout the body, including the central nervous system and importantly, the amygdala, and has an important role in both innate and adaptive immunity. It also promotes development of T helper 1 (Th1) cells in cell-mediated immune responses. In a past mouse study, it was revealed that the *IL18* gene in the amygdala increased susceptibility to chronic stress. Mice that were subjected to chronic stressors had increased expression of IL18 in the amygdala and exhibited depressive-like symptoms. Mice that had *IL18* knock-out were resilient to these stressors (92). The few human studies that have been conducted on this factor show similar results. One large study of 549 university students who completed the Duke Neurogenetics Study assessed resilience states through the Mini International Neuropsychiatric Interview. Amygdala reactivity to threat was also assessed using an emotional face matching challenge paradigm (in which the students viewed fearful, angry, surprised, and neutral faces) and functional magnetic resonance imaging (fMRI). It was identified via buccal cells that the *IL18* haplotype predicted increased threat-related reactivity of the left centromedial amygdala (93). This region contains the central nucleus of the amygdala, which results in the activation of the HPA axis in response to threat and stressors. The differences between the sexes of this particular genetic factor need to be explored, but it appears that lower expression of IL18 may be beneficial for resilience.

In a study of 96 adolescents (including 33 patients with psychotic disorders), fasting venous blood revealed that patients with early-onset psychosis had elevated levels of circulating IL18. They also had a higher ratio of IL18 to IL18BP (IL18’s binding protein), indicating a higher activity of IL18 in these patients and a more proinflammatory state overall (94). Although some studies fail to show an association with low resilience and IL18 levels. One previous study conducted on 3,012 participants diagnosed with PTSD demonstrated that compared to healthy controls, there was no association of higher IL18 levels with full PTSD, but those with the highest quartile of IL18 concentration was associated with partial PTSD (95). While proinflammatory states are the body’s natural response to stress, IL18 may be involved in too much increase in the stress response and therefore, lower levels of resilience.

### Tumor Necrosis Factor

*TNF* codes for tumor necrosis factor alpha (TNFα) and is also known as the master proinflammatory cytokine. This cytokine has been shown to play a vital role in the typical immune response through the regulation of a number of pathways encompassing an immediate inflammatory reaction with significant innate immune involvement as well as cellular activation with subsequent proliferation and programmed cell death or necrosis. TNFα levels have revealed correlations to many different disease states, ranging from autoimmunity to cancer (96). In a study with 88 healthy individuals participating in a sleep deprivation study, the TNFα 308A allele, which is less common than the 308G allele, was associated with greater resilience to psychomotor vigilance performance impairment during total sleep deprivation (regardless of time of day), and also provided a small performance benefit at baseline (97). In a cohort of trauma-exposed Vietnam War veterans (*n* = 299; 159 cases, 140 controls) TNFα serum levels and TNFα polymorphism rs1800629 were correlated with PTSD severity and resilience scores (81). In contrast, another study showed that lower plasma concentrations of the proinflammatory markers TNFα and IFNγ in the immediate aftermath of trauma are associated with greater risk for developing chronic PTSD symptoms (98). Overall, these results suggest individuals with PTSD show signs of a pro-inflammatory state as evidenced by higher levels of TNFα.

### C-Reactive Protein

C-reactive protein (CRP) has been discussed in a few resilience studies in association with other pro-inflammatory cytokines. One such study showed that among women, higher CRP was associated with increased severity on observed mood, cognitive symptoms, interest-activity, and suicidality, but these associations were not found in men (99). In another one, increased levels of proinflammatory markers including IL-6 and hsCRP were associated with lower psychological resilience and resilience scoring in PTSD patients (82). In contrast, one large population study demonstrated no association between full PTSD and CRP levels (95). Therefore, CRP is yet another example of a biomarker that shows mixed results in resilience studies and warrants further investigations.

## The Endocannabinoid System

Endocannabinoids, or endogenous cannabinoids, were measured in hair samples of EMS personnel and it was discovered that reported traumatic stress during childhood and later in life as well as more severe depressive and physical stress symptoms were associated with elevated 2-AG, SEA, OEA, and PEA concentrations (44). This contradicts other studies suggesting that concentrations of these molecules, such as 2-AG, were lower in individuals with PTSD that occurred as a result of the World Trade Center attacks (100). Additionally, significantly lower circulating 2-AG concentrations were also found in a study of individuals with PTSD as a result of childhood sexual abuse compared to controls (101). Mixed results suggest that dysregulation of endocannabinoids may be a sign of low resilience or PTSD, but further investigation is warranted to tease out the specifics.

## Future Directions and Resilience Interventions

All of the research on resilience, including the research in this review, is important in the development of future interventions of resilience. Many resilience interventions have been shown to have a positive effect on stress response and resilience, including cognitive behavioral therapy and mindfulness skills (102). These treatments could be especially useful in persons with stressful work conditions, such as first responders who routinely face trauma at work. A study of various interventions showed that those interventions that target specific modifiable risk factors of psychiatric disorders were the most beneficial in protecting the mental health of first responders (103). Stress inoculation is also a great tool used for professionals in fields such as this as well. However, there are no current interventions for resilience specifically that target genetics or biomarkers.

An example for the need of new resilience interventions is within major depressive disorder (MDD), a highly prevalent disorder affecting a significant amount of the world’s population. Currently there is a strong need for new medical interventions for MDD. Han and Nestler proposed that modifying susceptibility and increasing resilience to neural disorders could be the next therapeutic strategy. For example, they talked about the modification of the KCNQ channel, a K + channel expressed in the ventral tegmental area (VTA) and involved in the VTA-nucleus accumbens (NAc) pathway of the brain (the reward pathway). They proposed that giving channel openers (i.e., retigabine) for the KCNQ channel would actually help to treat depression, because in previous studies the KCNQ channel was upregulated in the VTA-NAc dopamine neurons of mice with high levels of resilience and low levels of depression (104). This is just an example of how targeting a physical biomarker of resilience may help in the development of treatments of neuropsychiatric disorders.

Understanding what makes an individual resilient is just the first step in developing preventative therapies. Many different approaches have been tested, including counseling and multifaceted interventions (105). Currently, there are studies of methods aimed at strengthening individuals’ resilience, especially methods of stress inoculation training. In this approach, participants are exposed to high-stress conditions or experiences (generally more moderate than similar real experiences), which are meant to prepare them for future stressors (106). The stress inoculation technique is already utilized in many high-stress job training programs, such as training for first-responders and medical staff. It also has been shown to be effective in other conditions as well, such as preventing PTSD in military members, anxiety and depression in cancer patients, or even to reduce exam anxiety in stressed-out students (107, 109). In the future, we will be able to more accurately monitor an individual for stress coping and resilience by combining information from self-reported measures, vital sign and demographic data, psychosocial, stress reactivity, genomics, proteomics, transcriptomics, metabolomics, and epigenetics to apply more of a comprehensive systems approach to diagnosis and treatment of stress induced disorders. For example, groups are already working on development and validation of an algorithm for prediction of post-traumatic stress disorder with promising results. Results demonstrate externally validated accuracy to discriminate PTSD risk with high precision (110). In this study, investigators combined measures of vetted mental health screening questionnaires, psychophysiological stress response, threat perception, psychophysiological arousal, immune and inflammatory markers and psychosocial determinants (110). More variables to consider here would be brain imaging, both structural and functional MRI, in response to stressful stimuli, with tissue specific epigenetic data and biomarker levels, in addition to the above-mentioned components. In the future, we will also be able to more accurately determine which stress inoculation or stress simulation tests are the most valuable, by continuing to monitor individuals engaged in these training sessions for stress biomarkers, stress reactivity and follow-up for mental health related disorders. This will allow development and optimization of the best training curriculum. In the meantime, members of trauma teams must stay committed to staying educated on how to best and make sure to build their own individual resilience.

Overall, it appears that any intervention to promote resilience is better than no intervention at all (111). Unfortunately, to our knowledge, therapies actually targeting physical biomarkers of resilience are extremely limited in real practice, and are still in the research stage. Perhaps these physical biomarkers could also help build algorithms for identifying people that could potentially suffer from a stress “break-down” or develop neuropsychiatric conditions associated with low resilience. We hope that this literature review of some of the psychological, genetic, epigenetic, and protein biomarkers involved with resilience will be the next step in developing more therapies that actually target the physical biomarkers of stress.

## Author Contributions

MR wrote the genetics and epigenetics sections of the manuscript, as well as the abstract and conclusion, and edited the manuscript and references. RR wrote the biomarker sections of the manuscript and contributed to the conclusion, and edited the manuscript and references. Both authors contributed to the article and approved the submitted version.

## Conflict of Interest

The authors declare that the research was conducted in the absence of any commercial or financial relationships that could be construed as a potential conflict of interest.

## Publisher’s Note

All claims expressed in this article are solely those of the authors and do not necessarily represent those of their affiliated organizations, or those of the publisher, the editors and the reviewers. Any product that may be evaluated in this article, or claim that may be made by its manufacturer, is not guaranteed or endorsed by the publisher.
